# Modeling Alzheimer's Disease as an Innate Immunity Persistent Activation Disorder

**DOI:** 10.1002/alz70855_102284

**Published:** 2025-12-23

**Authors:** Autumn Meek, Matthew P Neal, Donald F. Weaver

**Affiliations:** ^1^ Krembil Research Institute, Toronto, ON, Canada; ^2^ University Health Network, Toronto, ON, Canada

## Abstract

**Background:**

Developing a comprehensive molecular pathogenesis model for Alzheimer's (AD) is a research priority; to‐date, a range of different mechanistic proposals including proteopathy, immunopathy, gliopathy, synaptopathy, membranopathy, mitochondriopathy, oxidative stress, and metal dyshomeostasis have been proposed. Rather than unconditionally rejecting the role of any one specific disease mechanisms, the need for an innovative broadly‐encompassing model of AD, which harmonizes multiple divergent theories into a single unified comprehensive explanation, emerges as a much‐needed milestone on the road to a cure. Characterizing AD as an innate immunity mediated persistent neuroinflammatory disorder may provide such an all‐encompassing model.

**Method:**

We performed a comprehensive series of *in silico, in vitro* and *in vivo* studies explicitly evaluating multiple biochemical processes implicated in the pathogenesis of AD: Aβ/tau oligomerization, Aβ‐mediated membrane rupture, pro‐inflammatory cytokine release, mitochondrial damage, synaptotoxicity, and metal‐catalyzed reactive oxygen species generation. These analyses were then systematically probed for unifying mechanistic commonalities.

**Result:**

The following mechanistic model of AD was devised. Aβ has antimicrobial and immunomodulatory activities, functioning as a component of the innate immune system. In response to various stimuli (infection, trauma, ischemia, air pollution), Aβ is released as an early responder immunopeptide triggering an innate immunity cascade in which Aβ exhibits immunomodulatory and antimicrobial properties (whether bacteria are present, or not), resulting in a misdirected attack upon ‘self’ neurons, arising from analogous electronegative surface topologies between bacteria and neurons (particularly within the synaptic region), rendering them similarly susceptible to membrane‐penetrating attack by antimicrobial peptides such as Aβ. In its role as an antimicrobial‐immunomodulatory peptide, Aβ binds to monosialotetrahexosylganglioside (GM1) on the neuronal membrane surface to block viral entry, while intracellularly damaging mitochondria which are evolutionarily derived from endosymbiotic bacteria; concomitantly, Aβ binds to glial cells triggering release of neurotoxic pro‐inflammatory cytokines. Following these self‐directed attacks, the resulting neuronal breakdown products (particularly Aβ‐GM1 co‐aggregates) diffuse to adjacent neurons eliciting further release of Aβ, leading to chronic, persistent activation of innate immunity. AD thus emerges as a disorder of persistent innate immunity activation.

**Conclusion:**

A new unifying, comprehensive model of AD as an innate immunity persistent activation disorder has been devised.

